# Ulcerative colitis model triggers gut α-Synuclein aggregation without brain involvement or neuronal loss in female rats

**DOI:** 10.3389/fimmu.2025.1637548

**Published:** 2026-01-06

**Authors:** Ana María Espinosa-Oliva, María Dolores Vázquez-Carretero, Rocío Ruiz, María A. Roca-Ceballos, Pablo García-Miranda, María José Peral, Manuel Sarmiento Soto, Antonio J. Herrera, José Luis Venero, Rocío M. de Pablos

**Affiliations:** 1 Instituto de Biomedicina de Sevilla, IBiS/Hospital Universitario Virgen del Rocío/CSIC/Universidad de Seville, Sevilla, Spain; 2 Facultad de Farmacia, Departamento de Bioquímica y Biología Molecular, Universidad de Sevilla, Seville, Spain; 3 Facultad de Farmacia, Departamento de Fisiología, Universidad de Sevilla, Seville, Spain

**Keywords:** gut-brain axis, inflammation, neurodegeneration, Parkinson disease, sex differences, ulcerative colitis, α-Synuclein

## Abstract

**Introduction:**

Despite being the second most common neurodegenerative disorder, the mechanisms underlying the onset and progression of Parkinson’s disease (PD) remain poorly understood, and no curative treatment is currently available. The Braak hypothesis offers an intriguing framework for explaining both the origin and development of the disease, proposing that PD begins in the gut and subsequently spreads to the brain.

**Methods:**

In previous studies, our group developed a novel PD model in which peripheral inflammation, triggered by administering dextran sodium sulphate (DSS) in the drinking water of male Wistar rats, recapitulates key features of PD in both the gut and the brain. This model supports the Braak hypothesis and highlights the relevance of the gut-brain axis. Using the same model, the present study aimed to determine whether sex influences peripheral inflammation and the resulting neuropathology in the substantia nigra (SN) of female Wistar rats.

**Results:**

Our findings show that while DSS treatment induces comparable levels of colonic inflammation and phosphorylated α-synuclein accumulation in both sexes, it does not produce α-synuclein aggregation or dopaminergic neuronal loss in the SN pars compacta of female rats.

**Conclusion:**

These results underscore the critical importance of considering sex differences in experimental PD models and in clinical practice, as such differences may significantly influence PD pathogenesis.

## 1 Introduction

Parkinson’s disease (PD) is the second most common neurodegenerative disorder, surpassed only by Alzheimer’s disease. It is characterized by the selective loss of dopaminergic neurons in the substantia nigra pars compacta (SNpc) and the consequent reduction of dopamine in the caudate-putamen. This dopamine deficiency gives rise to the hallmark motor symptoms of the disease: rigidity, akinesia, tremor, and postural instability. These symptoms markedly diminish the quality of life of patients and their caregivers posing a substantial socioeconomic burden.

At the histopathological level, PD is defined by the accumulation of misfolded α-synuclein (α-syn), which aggregates into Lewy bodies (LB) within dopaminergic neurons of the SNpc (1). Multiple factors, including oxidative stress, mitochondrial dysfunction, reduced levels of trophic factors, alterations in the ubiquitin-proteasome system, and neuroinflammatory processes, are thought to interact in driving the progressive degeneration of SNpc neurons (2–4).

According to an analysis from the Global Burden of Disease, Injuries, and Risk Factors Study (GBD), PD is the fastest-growing neurological disorder in terms of prevalence, disability, and mortality (5). Over the course of the 20th century, PD has shifted from being considered a rare condition to becoming a major public health concern in developed countries. This rise is largely attributed to increasing life expectancy and the ageing population observed in recent decades.

For these reasons, it is essential to investigate and elucidate the underlying causes of PD onset, as well as the mechanisms driving disease progression, in order to develop new therapeutic strategies capable of counteracting neuronal loss. To this end, researchers are striving to develop the most appropriate animal models that faithfully recapitulate the key features of this complex disorder.

Accumulating evidence indicates that patients with ulcerative colitis (UC) may have an increased risk of developing PD (6–8). Consequently, the role of the gut–brain axis in PD pathogenesis has become a major focus of research in recent years. In this context, our research group recently published a novel PD model based on the induction of colonic inflammation that mimics human UC by administering dextran sulphate sodium (DSS) in the drinking water to male rats. This treatment induces severe gut inflammation accompanied by the accumulation of α-syn in the various layers of the colon and in the SN, along with a reduction in tyrosine hydroxylase (TH)-positive neurons in the SNpc, indicating dopaminergic neuronal loss (9). These findings provide strong support for the plausibility of Braak’s hypothesis and highlight the critical role of peripheral inflammation and the gut-brain axis in triggering α-syn aggregation and its propagation to the SN, ultimately leading to neurodegeneration.

Interestingly, PD exhibits a pronounced sex-related bias. Multiple studies, including data from the GBD, indicate a clear male predominance, with a male-to-female ratio of approximately 140:100 (5). This sex disparity is evident not only in disease prevalence but also in the severity of both motor and non-motor symptoms, as well as in the response to pharmacological and non-pharmacological treatments (10–12), with females generally presenting a less aggressive disease course. Given these differences, sex should be carefully considered in the development of new disease models.

In this study, using the UC model described above, we demonstrate that gut inflammation leads to phosphorylated α-syn accumulation in the colon but does not result in α-syn accumulation or neuronal loss in the SNpc of female rats, in contrast to our previous findings in males. These results underscore the critical importance of including representative samples of both sexes and, where applicable, the broader gender spectrum, in both experimental and clinical research to advance our understanding of the factors influencing the gut-brain axis.

## 2 Materials and methods

### 2.1 Animals and treatments

Female albino Wistar rats (200–300 g) were used in these experiments (4–10 animals per experimental group). Rats were housed at 22 ± 1°C and 60% relative humidity, under a 12-h light–dark cycle, with free access to food and water.

**Figure 1:**
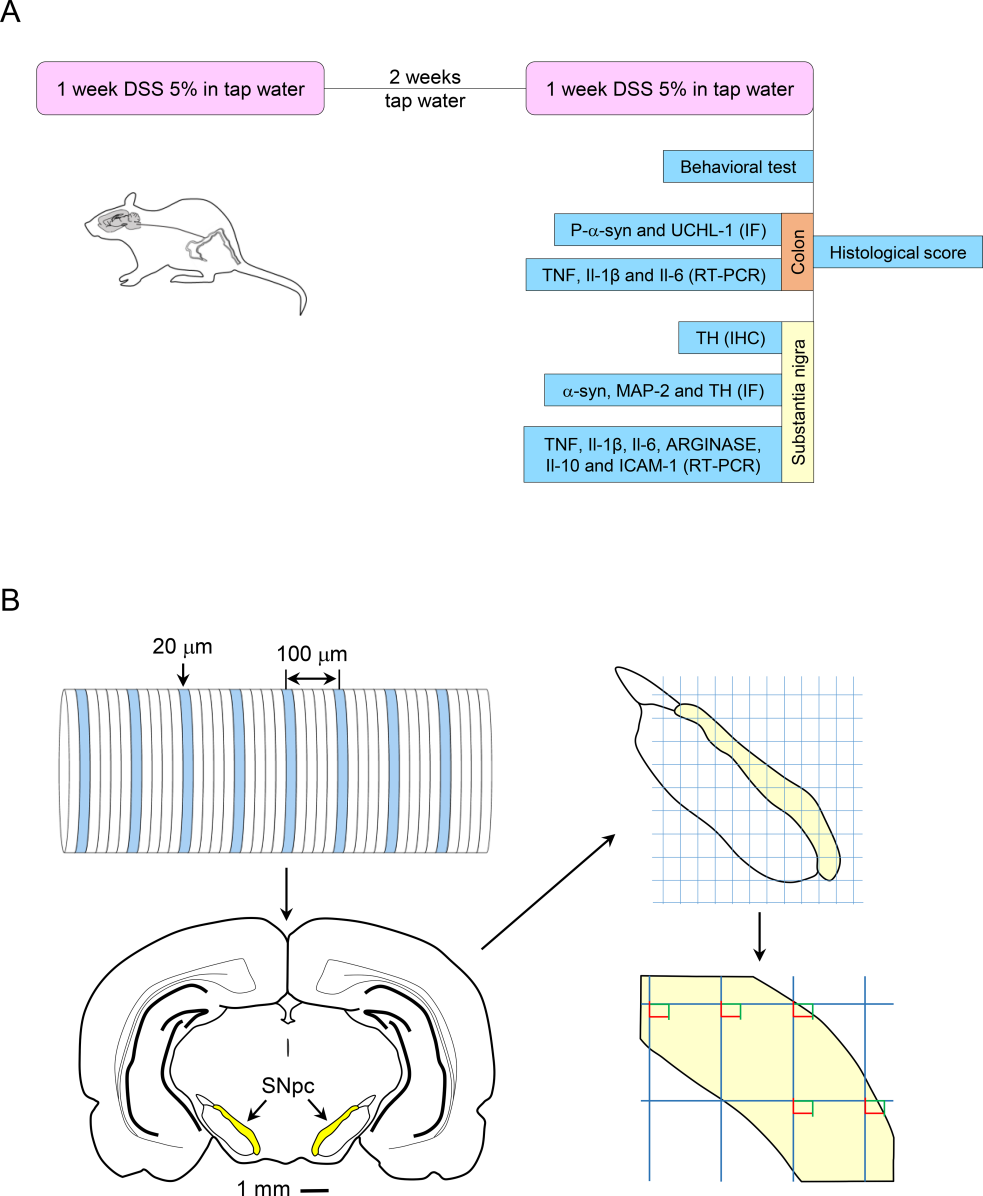
Timeline of DSS treatment in rats and schematic workflow for stereological counting of TH-positive neurons in the SNpc. **(A)** Experimental animals had free access to water containing 5% DSS (pink boxes) for two one-week periods, separated by a two-week interval during which they drank tap water. Control animals received only tap water. At the end of the treatment, various parameters were measured to assess the effect of subchronic colitis. **(B)** Animal brains were sectioned coronally at 20-µm thickness along the anteroposterior axis. Along the entire SN, schematized as a cylinder, one section out of every five (i.e., spaced 100 µm apart) was selected, with the first section chosen at random. The diagram in the lower left corner shows section 40 of the Paxinos and Watson (17). A grid measuring x = 150 µm and y = 200 µm was superimposed over the SN. The grid size was based on previous studies to satisfy the stereological criterion of counting at least 200 elements per animal, yielding an estimated coefficient of error of 7% according to the Poisson approximation. A counting frame of 40 µm x 25 µm was placed at the grid intersections within the SNpc. Cell counting followed the standard optical fractionator rules: cells entirely within the counting frame or touching the inclusion (green) lines were counted, while cells touching the exclusion (red) lines were excluded, even if partially within the frame. All criteria were predefined to minimize bias during counting.

We employed an experimental UC model based on the oral administration of DSS (molecular weight 36–50 kDa; PanReac AppliChem, Spain. Batch No. 9J013322) at a concentration of 5% (w/v) in drinking water. In this paradigm, female rats received DSS ad libitum for 1 week, following the method previously described by Okayasu (13). This was followed by 2 weeks of tap water and a final week of DSS administration, resulting in a total treatment duration of 28 days. Control animals received tap water throughout. A timeline of the DSS treatment and the parameters assessed is shown in **Figure 1A**.

Typical effects of DSS exposure, including rectal bleeding, diarrhea and weight loss, were evident by the end of treatment. In addition to these clinical signs, histopathological alterations in the gut resembled those observed in human UC. At the conclusion of the treatment period, animals were euthanized, and distal colons and brains were collected. The left and right hemispheres of each brain were analyzed separately. All animal procedures were conducted in accordance with the Guidelines of the European Union Directive (2010/63/EU) and Spanish regulations (BOE 34/11370-421, 2013) governing the use of laboratory animals. The study was approved by the Scientific Committee of the University of Seville.

The DSS treatment and subsequent measurements in female rats were conducted in parallel with those performed in male rats. Data obtained from the male cohort were previously published (9). Due to the severity of the UC-inducing treatment and in accordance with the 3R principles of replacement, reduction, and refinement, the study was not repeated in males. Instead, the previously published male data were used to calculate fold changes (FC DSS/water), which were then compared with the results from female rats to investigate potential sex differences.

### 2.2 Behavioral testing

The cylinder test is an ipsilateral-specific behavioral assay in which rats are placed inside a glass cylinder (19 cm in diameter, 35 cm in height) for 5 min without prior habituation. During spontaneous exploration, rats make repeated contacts with the cylinder walls using their forepaws. The number of wall touches made with each forepaw is recorded, and a right/left paw use ratio is calculated. All sessions were video-recorded for subsequent analysis. An imbalance in right versus left paw touches is indicative of ipsilateral motor impairment, making this test one of the most appropriate and reliable tools for detecting lateralized motor deficits, particularly those involving subtle hemispheric differences (14).

### 2.3 Histological analysis of colon

For histological assessment, paraffin-embedded sections of the distal colon were stained with hematoxylin/eosin. Analysis was performed in a blinded manner using a validated method (15). Colon damage was graded on a scale of 0–3 based on epithelial destruction, crypt dilatation, loss of goblet cells, inflammatory cell infiltration, oedema, and crypt abscesses. Each parameter was scored from 0 to 3, and the individual scores were summed to generate a cumulative damage score.

### 2.4 Immunohistological evaluation

Animals used for TH immunohistochemistry underwent 28 days of treatment before being perfused via the heart under deep anesthesia (isofluorane) with 150–200 ml of saline, followed by 150–200 ml of 4% paraformaldehyde in phosphate buffer (pH 7.4). After perfusion-fixation, brains were carefully removed and cryoprotected sequentially in sucrose solutions prepared in phosphate-buffered saline (PBS, pH 7.4): first in 10% sucrose for 24 h, and then in 30% sucrose until the brain sank (2–5 days). Brains were subsequently frozen in isopentane at -80°C. All incubations and washes were performed at room temperature (RT) in either Tris-buffered saline (TBS) or PBS, pH 7.4. Primary and secondary antibodies used are listed in **Table 1**. Coronal sections (20 µm) were cut on a cryostat at -20°C and thaw-mounted onto gelatin-coated slides. Sections were washed and then treated with 0.3% hydrogen peroxide in methanol for 20 min, followed by additional washes. Sections were then incubated in a humid chamber for 1 h in a blocking solution containing PBS, 0.1% Triton-X-100, 1% bovine serum albumin (BSA; Sigma), and 2% goat serum. Slides were drained and incubated with the primary antibody in a solution containing PBS, 0.5% Triton-X-100, 1% BSA, and 2% goat serum for 12 h at 4°C in a humid chamber. Following primary antibody incubation, sections were washed three times for 10 min each and then incubated with a biotinylated secondary IgG for 2 h. The secondary antibody was diluted in PBS containing 0.5% Triton X-100, 1% BSA, and 2% goat serum. Sections were subsequently incubated with VECTASTAIN^®^-Peroxidase solution (Vector; 1:100). Peroxidase activity was visualized using a standard diaminobenzidine (DAB)/hydrogen peroxide reaction for 5 min. Finally, slides were mounted in DPX (BDH Laboratories).

**Table 1:** Primary and secondary antibodies used for immunohistochemistry (IH) and immunofluorescence (IF).

Technique	1° antibody	Sample	Dilution	Company/batch number	2° antibody	Dilution	Company/batch number
IH	Rabbit-derived anti-TH	Rat brain	1:300	Sigma/SLBP1683V	Biotinylated goat anti-rabbit IgG	1:200	Vector/ZA0520
IF	Rabbit- derived anti α-syn	Rat brain	1:200	Santa Cruz Biotechnology/I0915	Alexa Fluor 594-conjugated anti-rabbit	1:500	Invitrogen/575274
IF	Mouse-derived anti MAP-2	Rat brain	1:200	Santa Cruz Biotechnology/E2419	Alexa Fluor 488-conjugated anti-mouse	1:500	Invitrogen/1890861
IF	Sheep-derived anti TH	Rat brain	1:500	Novus Biologicals/ajo419y	Alexa Fluor 647-conjugated anti-sheep	1:500	Invitrogen/1914541
IF	Rabbit-derived anti P-α-syn	Rat colon	1:300	Abcam plc/GR285618-1	Alexa Fluor 546-conjugated anti-rabbit	1:500	Invitrogen/1946340
IF	Mouse-derived anti UCHL-1	Rat colon	1:300	Abnova/HB231-55	Alexa Fluor 488-conjugated anti-mouse	1:500	Invitrogen/1890861

For immunofluorescence, animals also underwent 28 days of treatment before being perfused. Immunofluorescence staining for α-syn, MAP-2, and TH was performed on coronal brain sections. Sections were permeabilized with 1% Triton X-100 in PBS for 1 h, blocked with 5% (w/v) BSA and 1% Triton X-100 in PBS for 1 h, and then incubated overnight at 4°C with the primary antibodies diluted in 1% (w/v) BSA and 1% Triton X-100 in PBS. The following day, sections were rinsed for 1 h in PBS containing 0.1% Triton X-100 and subsequently incubated for 1 h with the corresponding secondary antibodies diluted in 1% (w/v) BSA and 0.1% Triton X-100 in PBS. Sections were then washed again in 0.1% Triton X-100 in PBS for 1h to remove excess of secondary antibodies. Finally, sections were counterstained with Hoechst 33258 (1:3000 dilution; Invitrogen) to visualize cell nuclei and mounted in 50% glycerol. Primary and secondary antibodies used are listed in **Table 1**.

For immunofluorescence detection of P-α-syn and UCHL-1 in distal colon samples, 5-μm-thick tissue sections were cut from paraffin blocks, dewaxed in xylene, and rehydrated through a series of graded alcohols. Sections were permeabilized by boiling in 0.01 M sodium citrate (pH 6) for 10 min. After blocking with 3% BSA, 3% fetal calf serum, and 0.1% Triton X-100 in PBS for 1h at RT, slides were incubated overnight at 4°C with the primary antibodies. Antibody binding was visualized using the appropriate fluorescent secondary antibodies. Nuclei were counterstained with Hoechst 33258 (1:3000 dilution; Invitrogen), and sections were mounted in 50% glycerol. Primary and secondary antibodies used are listed in **Table 1**.

### 2.5 Immunohistochemistry data analysis

For the analysis of immunofluorescence in colon sections, images were acquired using an Olympus BX61 microscope equipped with an Olympus DP73 camera. Quantification was performed using Image-J software. Five immunolabeled, non-consecutive, distal colon sections per rat were analyzed, with 9–15 fields examined per section to cover representative areas of each colon layer. An average of all measurements for each rat was calculated.

The number of TH-positive neurons in the SNpc was estimated using a fractionator sampling design (16). Because unilateral tremor is one of the earliest motor symptoms of PD, the left and right sides were analyzed separately. Counts were performed at regular predetermined intervals (x = 150 µm, y = 200 µm) within each section. The counted region spanned a thickness of 840 µm, corresponding to plates 38–42 in the atlas of Paxinos and Watson (17). Systematic sampling of the SNpc area was initiated from a random starting point. An unbiased counting frame of known area (40 µm x 25 µm = 1000 µm^2^) was superimposed on each tissue section image under a 60x objective, yielding an area sampling fraction of 1000 µm^2^/(150 µm x 200 µm) = 0.033. The entire z-dimension of each section (20 µm for all animals) was sampled, giving a section thickness sampling fraction of 1. Sections spaced 100 µm apart were analyzed (one section out of every five), resulting in a fraction of sections sampled of 20/100 (0.20). **Figure 1B** shows a schematic of the process. The total number of TH-positive neurons in the SNpc was estimated by multiplying the number of neurons counted within the sample regions by the reciprocals of the area sampling fraction and the fraction of sections sampled. To extrapolate the total number of neurons in the structure from the sampled volume using the dissector method, the software applies the Cavalieri principle, ensuring an unbiased final estimate.

For immunofluorescence quantification in the SNpc, the fluorescence intensity of TH-positive neurons was analyzed in six regions of interest (ROIs), three per hemisphere, in sections from six control females and seven females subjected to the DSS model. Images were acquired using an inverted ZEISS LSM 7 DUO confocal laser scanning microscope under identical laser intensity and gain settings. All images were captured at the same Z-plane to ensure consistent optical depth. Quantification was performed using ImageJ software (public domain: http://rsb.info.nih.gov/ij/download.html) without any additional image processing. To control for potential variability in tissue permeability across sections, Hoechst staining was also analyzed.

### 2.6 Real-time quantitative reverse transcription PCR

Left and right SN and distal colon samples were dissected from each rat after 28 days of treatment, snap-frozen in liquid nitrogen, and stored at −80°C. Total RNA was extracted using the RNeasy^®^ kit (Qiagen). Complementary DNA (cDNA) was synthesized from 1 μg of total RNA using the QuantiTect^®^ reverse transcription kit (Qiagen) in 20 μl reaction volume, following the manufacturer instructions. Real-time PCR was performed using iQ™SYBR^®^ Green Supermix (Bio-Rad Laboratories) with 0.4 μM primers and 1 μl of cDNA. Negative controls were run without cDNA. Amplification was carried out on a Mastercycler^®^ ep realplex (Eppendorf) thermal cycler with an initial denaturation at 94°C for 3 min, followed by 35 cycles of 94°C for 30 s, 55°C for 45 s, and 72°C for 45 s, and a final elongation step at 72°C for 7 min. A melting curve analysis was performed immediately afterward by heating the reactions from 65 to 95°C in 1°C increments while monitoring fluorescence, confirming the presence of a single PCR product at the expected melting temperature. β-actin served as the reference gene for normalization. Primer sequences for IL-1β, TNF, IL-6, ICAM-1, arginase, IL-10, and β-actin are provided in **Table 2**. The cycle threshold (Ct) for each sample was determined, and the triplicate values for each cDNA were averaged. Relative quantification was performed using the comparative Ct method integrated into the Bio-Rad System Software.

**Table 2:** Primers used for real-time PCR.

ARNm target	Primers sense (S) and antisense (A) sequences	Temp (°C)
β-Actin	S:5´-TGTGATGGTGGGAATGGGTCAG-3´ A:5´-TTTGATGTCACGCACGATTTCC-3’	60
IL-1β	S:5´-CAGGATGAGGACATGAGCACC-3´ A:5´-CTCTGCAGACTCAAACTCCAC-3´	60
TNF	S: 5’-TACTGAACTTCGGGGTGATTGGTCC-3’ A:5’-CAGCCTTGTCCCTTGAAGAGAACC-3’	60
IL-6	S:5´-AAAATCTGCTCTGGTCTTCTGG-3´ A:5´-GGTTTGCCGAGTAGACCTCA-3´	60
ICAM-1	S:5´-GACCACGGAGCCAATTTCTC-3´ A:5´-TCTTGAACAGTGACAGCCTTC-3´	60
Arginase	S: 5’-CCACGGTCTGTGGGAAAAGCCAAT-3’ A: 5’-TTGCCATACTGTGGTCTCCACCCA-3’	60
IL-10	S: 5’-TAAGGGTTACTTGGGTTGCC-3’ A: 5’-GTATCCAGAGGGTCTTCAGC-3’	60

### 2.7 Statistics

Statistical analyses were performed using GraphPad Prism 8.4.0. Prior to analysis, all data were assessed for normality using the Shapiro-Wilk test; some datasets passed the normality test, while others did not. To ensure consistency, comparisons were performed using either Mann-Whitney U test for two independent groups or two-way ANOVA followed by Sidak’s multiple comparisons test for analyses involving more than two groups. Sidak’s test includes a correction for multiple comparisons. Data are presented as mean ± standard deviation (SD). The study of statistical power and n-value was performed using the G*Power 3.1.9.4 software.

**Figure 2:**
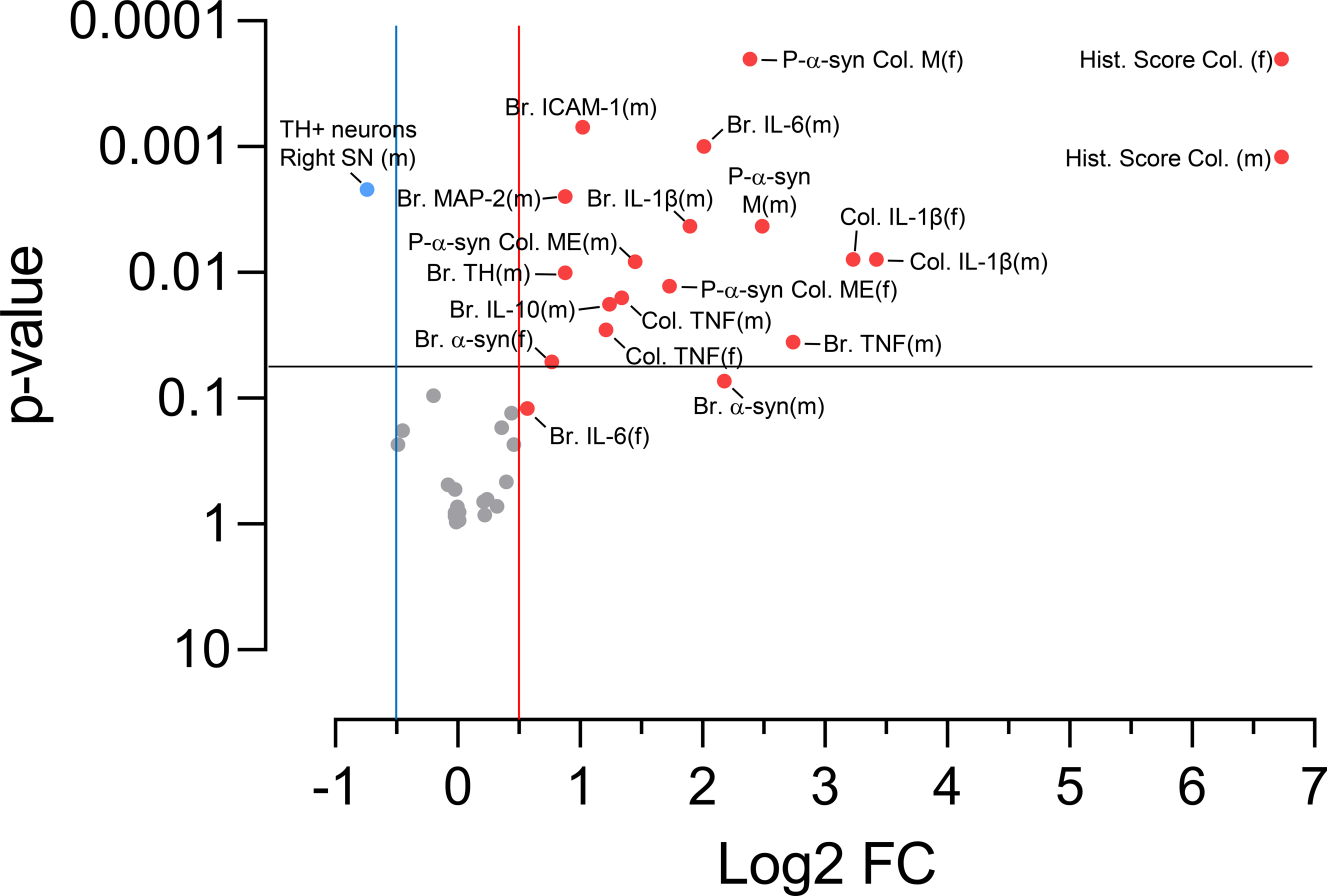
Volcano plot of the fold change (FC, DSS/water) of females and males. The x-axis represents the Log2 FC and the y-axis represents the –Log_10_ p-value. Significantly increased FCs (p <0.05, horizontal black line) with Log_2_ FC > 0.5 are shown in red, and significantly decreased FCs (p <0.05, Log2 FC < -0.5) are shown in blue. No significant FCs are shown in gray. (f), females; (m), males; Br., parameter measured in brain; Col., parameter measured in colon; M, mucosa; ME, muscularis externa.

To calculate the FC, the average of the control group (water) and the treated group (DSS) is found, and the treated average is divided by the control average. The Log2 of the FC has also been calculated. **Figure 2** shows a volcano plot summarizing the statistical significance of the FCs described throughout the text.

The statistical significance of each FC (DSS/water) was assessed using the Mann-Whitney U test. Differences in FCs between females and males were studied using two-factor ANOVA, where the factors were sex and treatment. The level of statistical significance of the interaction between these two factors indicates whether there are differences between the FCs of females and males for each parameter studied.

The study of statistical power and n-value was performed using the G*Power 3.1.9.4 software. Due to the severity of the UC-inducing treatment and in accordance with the 3R principles of replacement, reduction, and refinement, the number of animals per group was minimized taking into account the effect size observed for the different parameters in our previous study in males. The statistical power of the analysis of the parameters showing a significant difference between the control and DSS-treated animals ranged from 0.75 to 1. The parameters without statistical significance, showing a very small effect size (Cohen’s d), showed low powers (between 0.05 and 0.67). The *a priori* study using Cohen’s d to calculate the n-value needed to achieve a power of 0.8 for these parameters yielded values so high (hundreds of animals) that they cannot be used.

## 3 Results

### 3.1 Female rats develop UC in a manner similar to male rats

**Figure 3:**
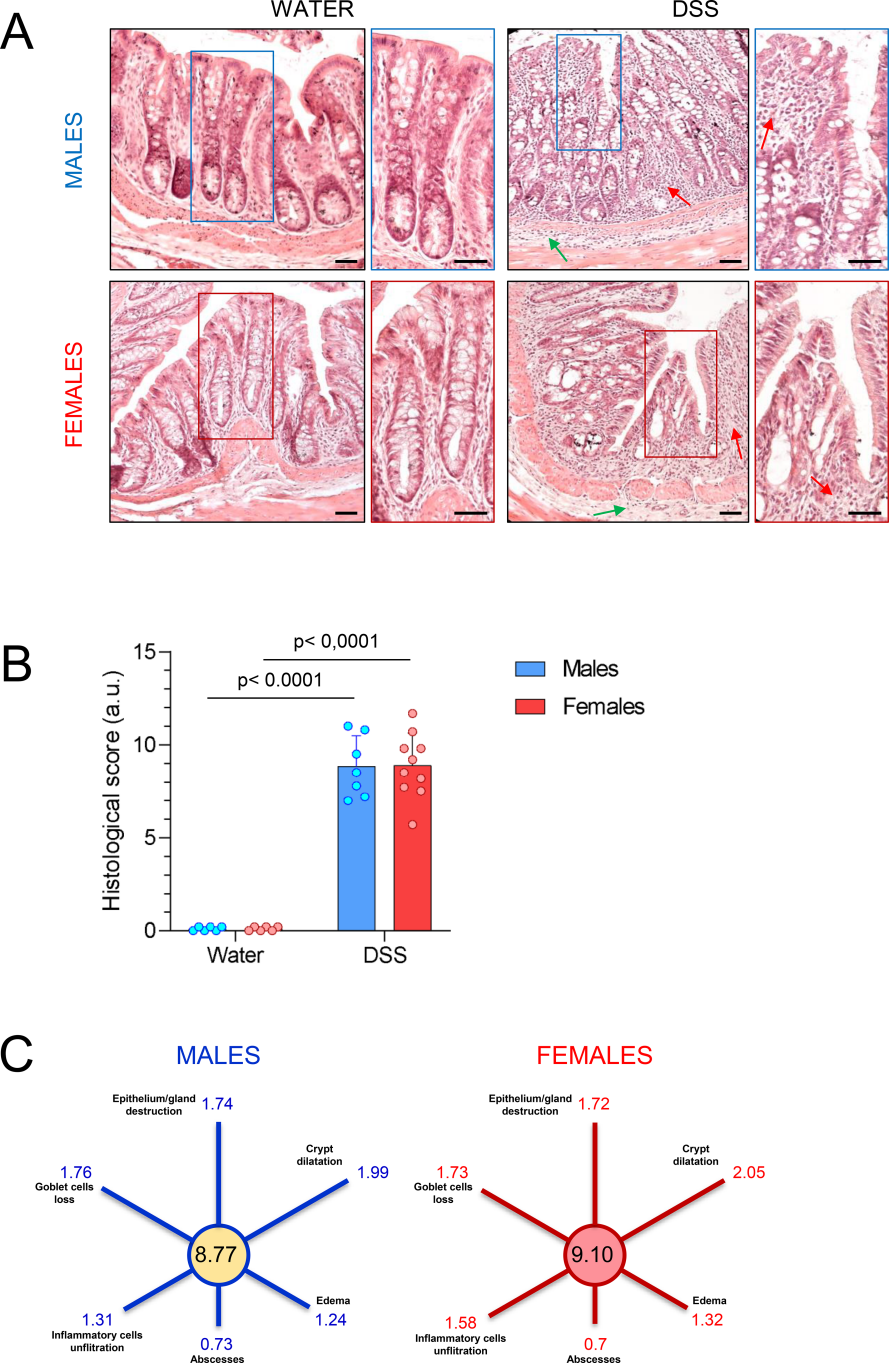
Histological analysis of the colon from DSS-treated and untreated female and male rats. **(A)** Representative hematoxylin-eosin-stained sections of the distal colon from water- and DSS-treated groups. The region outlined by the dotted square is shown at higher magnification beside each image. Inflammatory cell infiltration is indicated by red arrows in the mucosa and green arrows in the submucosa. Scale bar:100 μm. **(B)** Total colon damage scores in male and female rats. Statistical analysis: Mann-Whitney test for independent samples, α=0.05; p< 0.0001 for DSS-treated versus control (water) groups (n = 6–10 animals per group). **(C)** Mean scores for male and female rats treated with DSS across the six parameters used to assess colon damage.

Female rats received DSS or normal drinking water according to the experimental design illustrated in **Figure 1A**. First, we aimed to determine whether the experimental UC model induced inflammation and damage in the colon of female rats and whether the response differed from that in males (data previously published in (9)). To assess this, histological analysis was performed on distal colon sections stained with hematoxylin and eosin from DSS-treated and untreated (control) female rats. A total histological score was calculated as the sum of six parameters (each scored from 0 to 3, 0 = healthy, 3 = severely damaged): epithelial/glandular destruction, crypt dilatation, goblet cells loss, inflammatory cell infiltration, edema, and crypt abscesses. Representative images in **Figure 3A** illustrate the loss of crypt architecture and infiltration of inflammatory cells in the mucosa and submucosa of the colon in both female and male DSS-treated rats. Rats with colitis exhibited significant colonic damage compared to controls, as indicated by an increased total histological score (**Figure 3B**; p < 0.0001; n = 6-10. See also **Figure 2** for the significance of the FCs), with no difference observed between sexes (**Figures 3B, C**). Analysis of each parameter individually (**Figure 3C**) further demonstrated that DSS treatment induced comparable colon injury in male and female rats.

**Figure 4:**
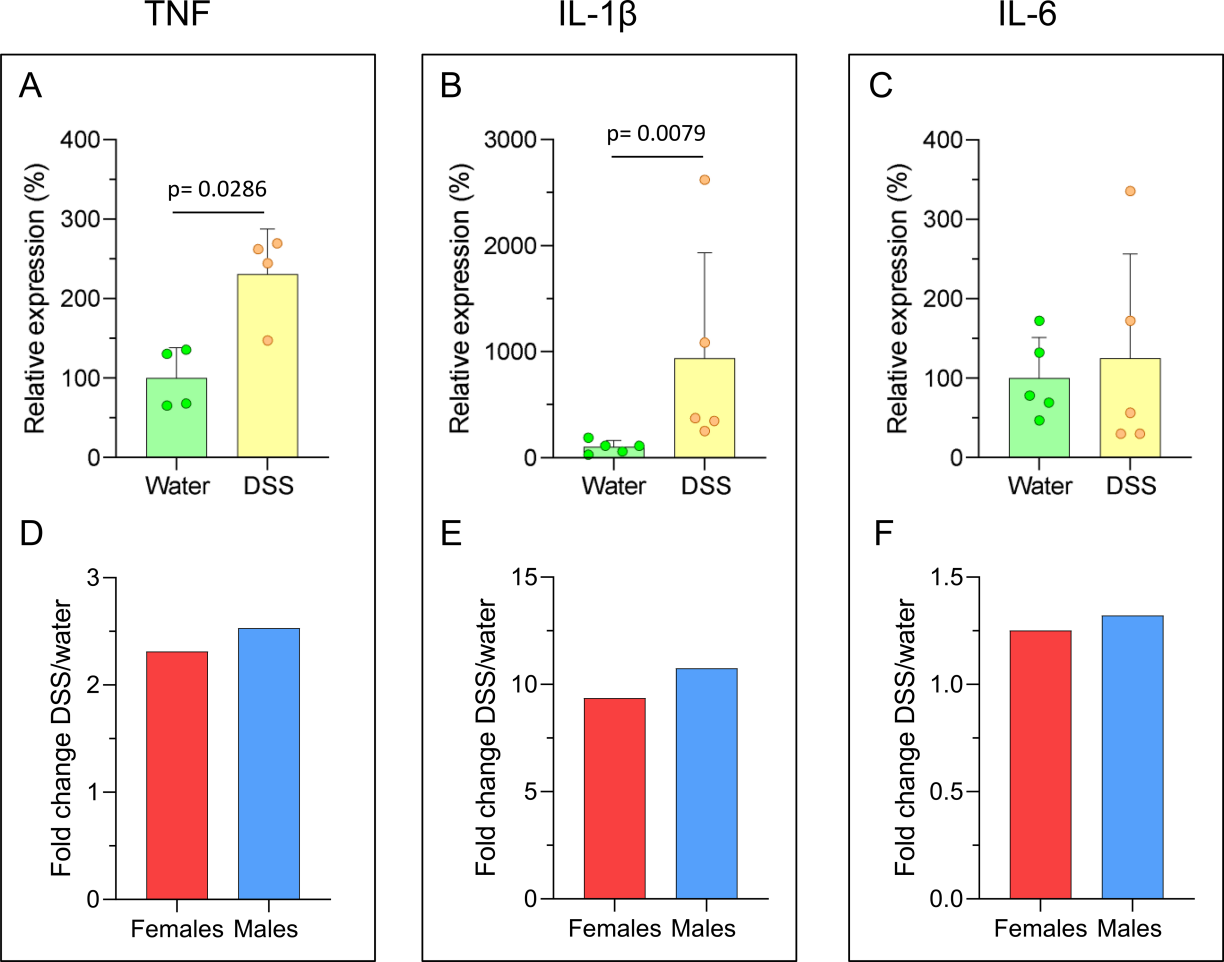
mRNA relative expression of pro-inflammatory cytokines in the colon of DSS-treated and control rats. Real-time quantitative reverse transcription PCR (RT-qPCR) was used to measure the expression of **(A)** tumor necrosis factor (TNF), **(B)** interleukin (IL)-1β, and **(C)** IL-6 in the distal colon of control (water) and DSS-treated female rats. Results are expressed as a percentage of the control group. **(D-F)** Fold-changes values for male and female rats are shown. Statistical analysis: Mann-Whitney U test for independent samples (α=0.05), comparing DSS-treated versus control (water) groups [**(A-C)**; n = 4–5 animals per group]; two-factor ANOVA for differences between the FCs of females and males.

In addition, we assessed the relative mRNA expression of the pro-inflammatory cytokines tumor necrosis factor (TNF), interleukin (IL)-1β, and IL-6 in the colon of these animals (n= 4-5) using RT-qPCR. In female DSS-treated rats, TNF expression was significantly increased (231.0% of controls, p= 0.0286; **Figure 4A**), as was IL-1β expression (935.8% of controls, p= 0.0079; **Figure 4B**); whereas IL-6 expression showed no significant changes (**Figure 4C**; See also **Figure 2**). The comparison of FCs of the relative expression of cytokines showed no significant sex differences (**Figures 4D-F**).

### 3.2 UC triggers accumulation of phosphorylated α-syn in the gut of female rats

**Figure 5:**
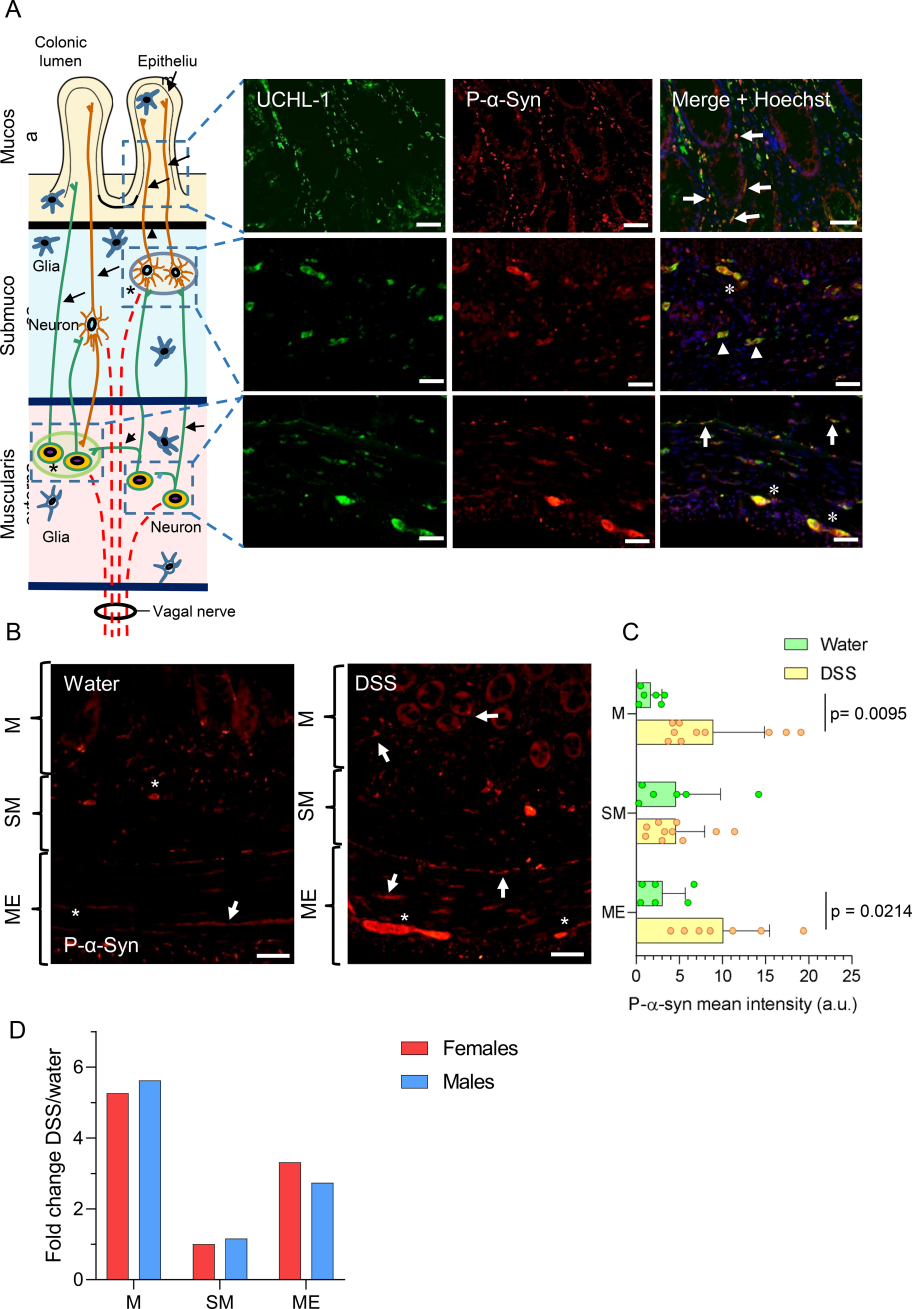
Immunolocalization of P-α-syn in the colon of female rats under subchronic DSS treatment. **(A)** Schematic cross-section of the distal colon wall illustrating the interconnected enteric plexuses, which contain neurons projecting to the mucosa, as well as vagal fibers. Immunofluorescence co-localization of UCHL-1 and P-α-syn is shown in nerve fibers (arrows), ganglia (asterisks), and neuronal somas (arrowheads) within the colon layers of a DSS-treated female rat. **(B)** Representative images of P-α-syn staining in the mucosa (M), submucosa (SM), and muscularis externa (ME) of control (water) and DSS-treated female rats. Neuronal structures are indicated as in **(A)**. Scale bars: 20 μm **(A)**, 50 μm **(B)**. **(C)** Quantification of P-α-syn fluorescence intensity in arbitrary units (a.u.) in the mucosa (M), submucosa (SM), and muscularis externa (ME). **(D)** Fold-change values for male and female rats. Statistical analysis: two-way ANOVA followed by Sidak’s multiple comparisons test (α=0.05) comparing DSS-treated versus control (water) groups (panel **C**, n = 6–10 animals per group); two-factor ANOVA for differences between the FCs of females and males **(D)**.

We next investigated whether our UC model induces pathological α-syn aggregates in the colon. Immunofluorescence analysis of distal colon sections was performed to assess the accumulation of phosphorylated α-syn (P-α-syn) in female DSS-treated rats. This region was selected because DSS-induced pathology is largely restricted to the distal colon, and its histological features closely mirror those observed in human UC. Co-localization of P-α-syn with the pan-neuronal marker ubiquitin C-terminal hydrolase (UCHL)-1 demonstrated the presence of P-α-syn within the enteric nervous system, including both the myenteric and submucosal plexuses of rats with UC (**Figures 5A, B**). P-α-syn was detected in mucosal nerve fibers surrounding the crypts, as well as in neuronal somas and fibers within the submucosal and muscular externa layers. P-α-syn immunoreactivity was also observed in non-neuronal cells, likely of immune or glial origin. Quantification of fluorescence intensity (n = 6-10) revealed a significant increase in P-α-syn signal in the mucosa (523%; p= 0.0095) and muscularis externa (326%; p= 0.0214) of DSS-treated female compared with controls, but not in the submucosa (100%) (**Figure 5C**; see also **Figure 2**). FC analysis (DSS/water) performed for each colon layer showed the same pattern in male rats, with no sex-related differences (**Figure 5D**).

### 3.3 UC did not induce microglial activation in the substantia nigra of female rats

**Figure 6:**
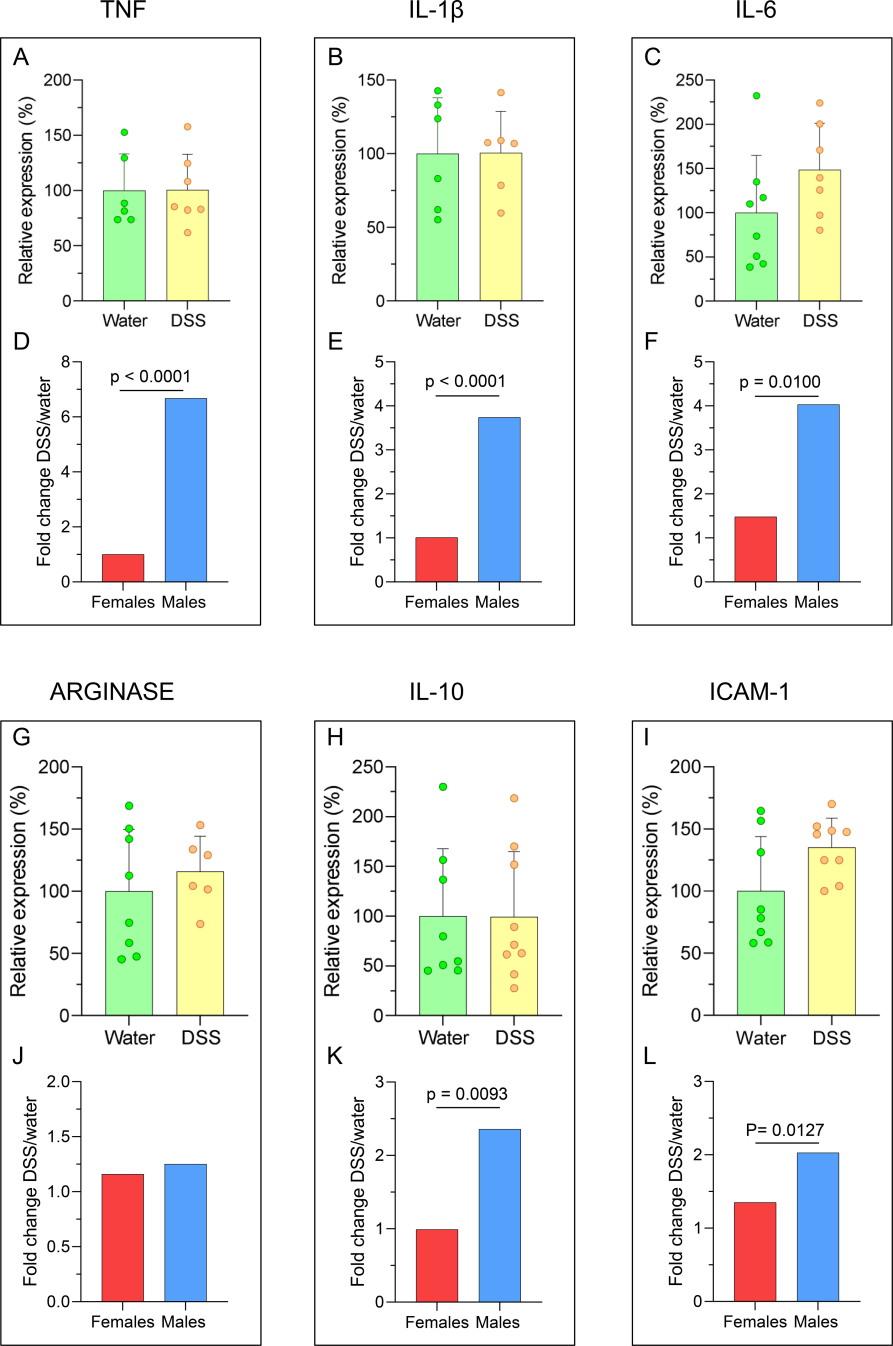
Expression of pro- and anti-inflammatory cytokines and intercellular adhesion molecule (ICAM)-1 in the SN of DSS-treated and control rats. Real-time quantitative reverse transcription PCR (RT-qPCR) was used to measure the expression of **(A, D)** tumor necrosis factor (TNF), **(B, E)** interleukin (IL)-1β, **(C, F)** IL-6, **(G, J)** arginase, **(H, K)** IL-10 and **(I, L)** ICAM-1 in the brain of control (water) and DSS-treated female rats. Results are expressed as a percentage of the control group. FC values for male and female rats are shown **(D-F, J-L)**. Statistical analysis: Mann-Whitney U test for independent samples (α=0.05) comparing males and females [panels **(A-C)** and **(G-I)**, n=6–9 animals per group]; two-factor ANOVA for differences between the FCs of females and males **(D-F, J-L)**.

Because our DSS treatment induces inflammatory signs and symptoms, as well as the accumulation of toxic α-syn aggregates in the colon of female rats, we next investigated whether this peripheral inflammation could extend to the SN, as previously reported in males (9). To assess neuroinflammation in the SN of DSS-treated females, we performed RT-qPCR for several pro- and anti-inflammatory cytokines, including the pro-inflammatory markers TNF, IL-1β, and IL-6 (**Figures 6A-C**), and the anti-inflammatory markers arginase-1 and IL-10 (**Figures 6G, H**). We also examined expression of the intercellular adhesion molecule (ICAM)-1 due to its role in immune-cell infiltration (**Figure 6I**). No significant differences in the expression of any of these genes (n = 6-9) were detected compared with water-treated controls. FC (DSS/water) for mRNA expression in female rats were non-significant (**Figure 2**).

However, FC values were consistently higher in males (**Figure 2**) than in females across all markers examined (**Figures 6D-F, K, L**), with the exception of arginase-1 (**Figure 6J**), which showed no sex-related difference.

### 3.4 Dopaminergic cells are not affected by DSS treatment in female rats

**Figure 7:**
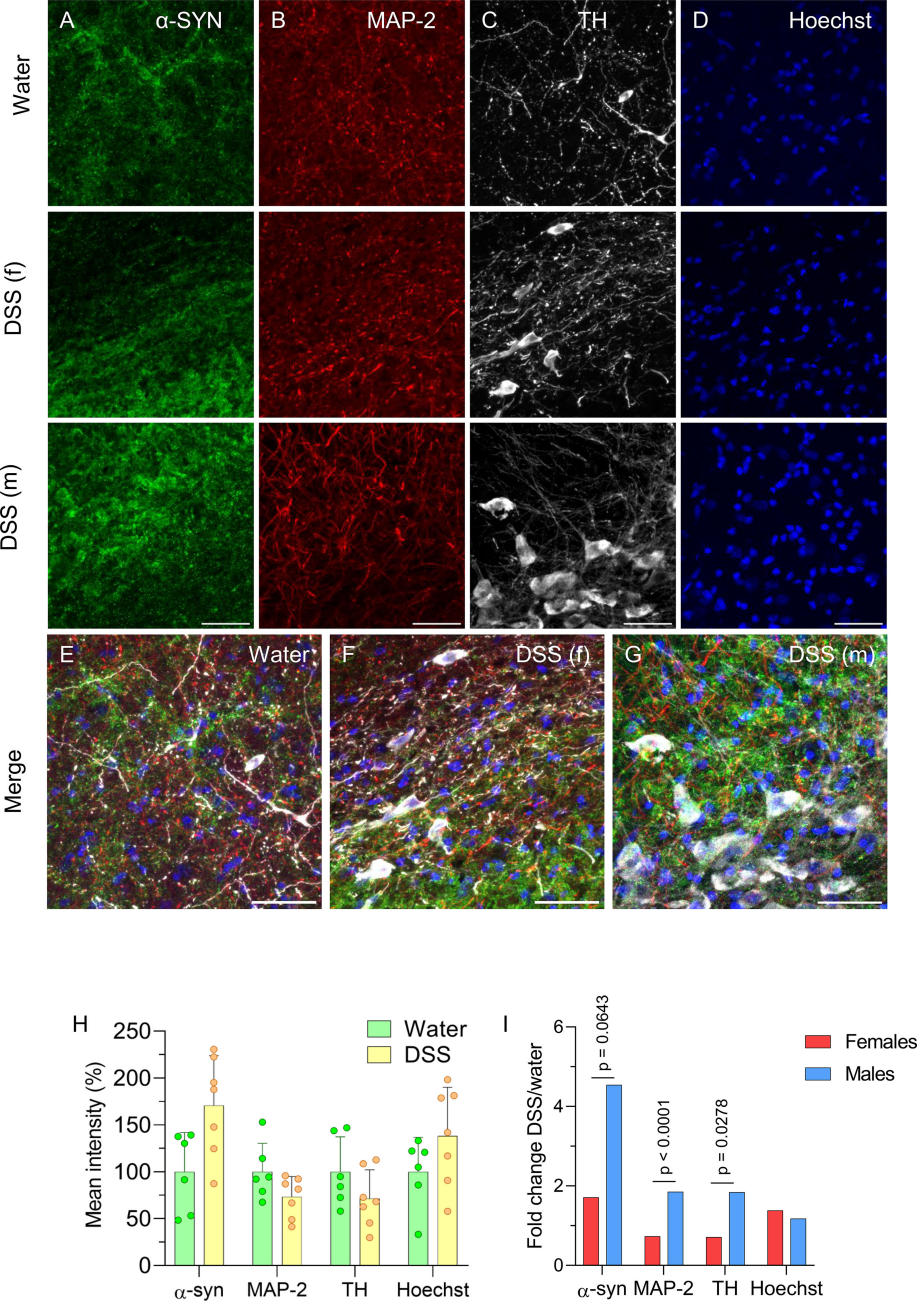
Expression of α-synuclein (α-syn), microtubule-associated protein (MAP)-2, tyrosine hydroxylase (TH), and Hoechst staining in the SN of DSS-treated and control rats. Coronal brain sections showing immunofluorescence staining for α-syn (column **A**), MAP-2 (column **B**), TH (column **C**), and Hoechst (column **D**) in the SN of control (water, first row), DSS-treated female (f) rats (second row) and DSS-treated male (m) rats (third row); merge images are shown in the fourth row **(E–G)**. Scale bar: 50 µm **(H)** Quantification of the markers shown in panels **(A-D)**; results are expressed as the percentage of mean fluorescence intensity relative to the control (water) (n = 5–7 animals per group). **(I)** Fold-change values for male and female rats. Statistical analysis: Mann-Whitney U test for independent samples (α=0.05) comparing males and females (panel **H**, n = 5–7 animals per group); two-factor ANOVA for differences between the FCs of females and males **(I)**.

We performed immunofluorescence analyses on brain sections to assess the intensity of α-syn, microtubule-associated protein (MAP)-2, and TH staining in the SNpc of female DSS-treated and control rats, and found no significant differences (**Figures 7A-C, E-H**). As a control for potential tissue-processing variability, we quantified Hoechst mean fluorescence intensity across all sections (**Figure 7D**), which showed no significant differences (**Figure 7H**; n = 6-7). FC analysis revealed lower MAP-2 (39.5%) and TH (38.6%) signal intensities in female rats compared with males (**Figure 7I**; see also **Figure 2**).

**Figure 8:**
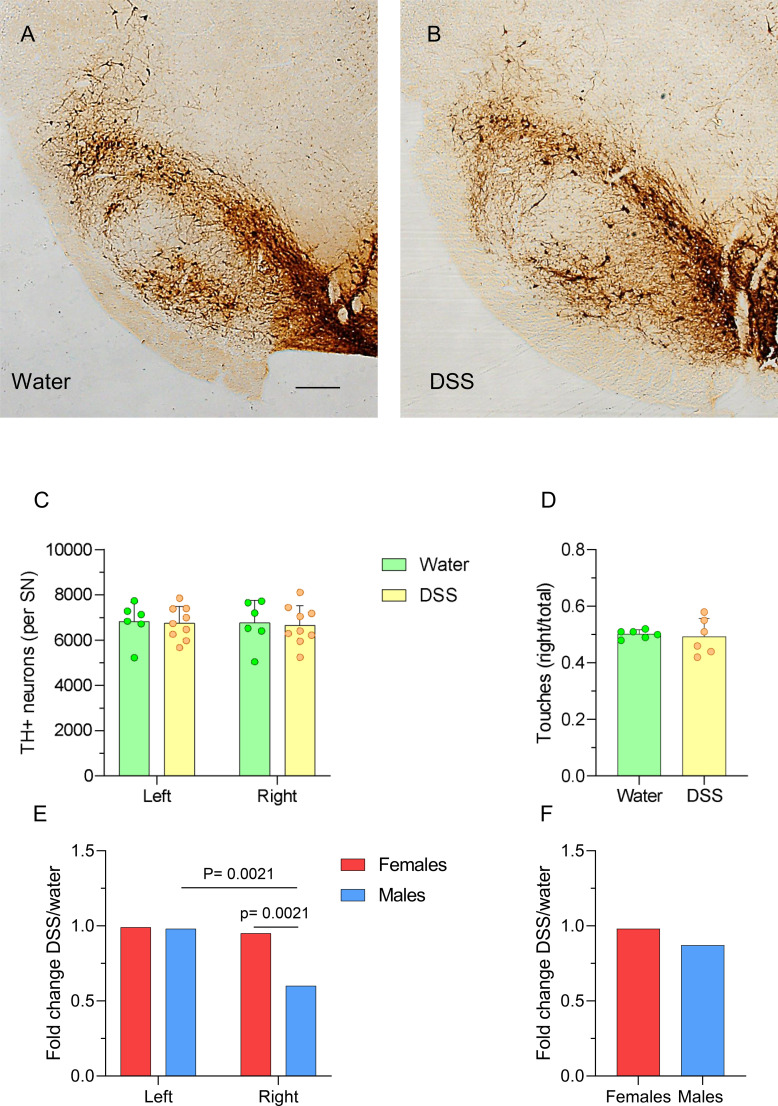
Dopaminergic neurons in the SN of DSS-treated and control rats. Coronal brain sections showing tyrosine hydroxylase (TH) immunostaining in the SN of control **(A)** and DSS-treated **(B)** female rats. Scale bar: 1 mm. **(C)** Stereological quantification of TH-positive neurons; results are expressed as the number of TH-positive neurons per SN (n = 6–9 animals per group). Error coefficients, estimated using the Poisson approximation (1/√number of neurons counted per animal), range from 0.05 to 0.064 (5.0 to 6.4%). **(D)** Motor performance of control and DSS-treated rats assessed using the cylinder test; results are expressed as the ratio of right to total forelimb touches (n = 6 animals per group). **(E, F)** FC values for female and male rats. Statistical analysis: Two-way ANOVA followed by Sidak’s multiple comparisons test **(C, E, F)**, and Mann-Whitney U test for independent samples (α=0.05); comparing males and females **(D)**.

We also employed an unbiased stereological approach to quantify TH-positive neurons in the SNpc of female animals following DSS treatment. The analysis revealed no significant differences between DSS-treated and control groups (control: 6823 ± 860 and 6762 ± 1005 TH-positive cells in the left and right SNpc, respectively; DSS: 6752 ± 723 and 6656 ± 870 TH-positive cells in the left and right SNpc, respectively; **Figures 8A-C**; n = 6-9). These findings are consistent with motor-behavior assessments using the cylinder, which showed no significant differences between DSS-treated and control females (**Figure 8D**; n = 6).

FC analysis showed no differences in behavioral performance (**Figure 8F**; n = 6; see also **Figure 2**). However, it revealed a significant reduction of TH-positive neurons in the right SNpc of males (40%, p = 0.0021; **Figure 8E**; n = 6-9).

## 4 Discussion

In this study, we demonstrate that DSS administration induces subchronic colitis in female rats that closely mimics human UC. We observe P-α-syn accumulation in the gut, but no evidence of neuroinflammation or dopaminergic neuron loss in the SNpc.

The idea that peripheral inflammation can increase the vulnerability of the nigrostriatal dopaminergic system is well established. Several studies have shown that peripheral inflammatory processes can exacerbate dopaminergic neurodegeneration in different PD models (11, 18–20). In this context, our group recently reported a novel PD model driven by peripheral inflammation associated with DSS-induced subchronic colitis in male rats (9). That study demonstrated that UC in males led to the formation of P-α-syn inclusions in the Auerbach and Meissner plexuses of the gut, as well as increased α-syn expression and dopaminergic neurodegeneration in the SNpc, two key hallmarks of PD (21). An advantage of this model is that it avoids direct manipulations of the SN, such as toxin delivery or transgenic overexpression of α-syn, thereby providing a more physiological system that still recapitulates major histopathological features of the disease. These findings strongly support the plausibility of Braak’s hypothesis and highlight the importance of peripheral inflammation and the gut-brain axis in initiating α-syn aggregation and its subsequent propagation to the SN, ultimately contributing to neurodegeneration.

As noted above, PD exhibits a marked sex-related bias, with men being approximately 1.4 times more likely than women to develop the disease. Sex differences are also evident in symptom onset, disease progression, and treatment responses. For example, men and women differ in several prodromal markers, including subthreshold parkinsonism, constipation, olfactory loss, and depression (22). Consequently, there is an increasing need for comparative studies in diverse animal models of PD to clarify the biological basis of these sex differences and to inform the development of more effective therapeutic strategies.

In this study, we examined the effects of oral DSS treatment on the colon and assessed the presence of α-syn accumulations in both the colon and SNpc of female rats. Analyses of α-syn staining in colon were performed in the distal region, which was sufficient to detect significant colitis-associated changes. However, this approach may limit the ability to evaluate alterations along the entire length of the colon. Future studies employing Swiss-roll preparations would allow a more comprehensive assessment and help determine whether these effects are uniformly distributed throughout the colon. We also evaluated the impact of DSS treatment on the integrity of dopaminergic neurons in the SNpc. Notably, after DSS exposure, the distal colons of female rats displayed marked inflammation, histological damage, and elevated proinflammatory cytokine mRNA levels, findings consistent with those previously reported in males (9). Several studies have described conflicting observations regarding sex differences in DSS-induced outcomes (23–25), likely reflecting variability in experimental paradigms, including DSS concentration and exposure duration. In our UC model, we did not detect any sex-dependent differences in colitis severity.

Additionally, P-α-syn expression increased in the distal colons of DSS-treated female rats, displaying the same localization patterns previously observed in males (9). However, in contrast to DSS-treated male, females showed no evidence of neuroinflammation, α-syn aggregation, or dopaminergic neuronal loss in the SNpc, underscoring a clear sex-dependent difference in disease progression. Although additional behavioral assays (e.g., rotarod or open-field test) could further support these findings, no differences were detected using the cylinder test. Overall, these results demonstrate a pronounced sexual dimorphism in this colitis-induced PD model, in which males develop parkinsonian pathology whereas females do not.

The reasons for this discrepancy between males and females are likely multifactorial and may influence several of the mechanisms implicated in PD pathogenesis. A substantial body of literature documents clear sex-related differences in the human brain (reviewed in (22), and numerous studies indicate that females are generally more resistant to neurodegeneration (26–30). Sex differences in PD animal models have also been comprehensively reviewed (31–33).

Interestingly, gene expression studies of dopaminergic neurons in the human SNpc, conducted in both control subjects and PD patients, have identified a sex-dependent genomic signature. Genes upregulated in females are predominantly associated with signal transduction and neuronal maturation, whereas genes upregulated in males encode proteins linked to disease pathogenesis, such as α-syn or PINK1 (34, 35).

Sex-dependent differences in dopamine metabolism are well documented. For example, the density and binding potential of striatal dopamine D2 receptors decline approximately twice as rapidly in aging men (36), whereas women generally have a higher number of dopaminergic neurons and greater striatal dopamine uptake (37). Additionally, women show a predominance of D1 receptors, which may contribute to increased resilience against certain neurological disorders (38). In contrast, the SRY gene on the Y chromosome may exert specific effects on dopaminergic neurons in men (39). Collectively, these differences suggest that dopaminergic neurons in females are often less vulnerable to neurodegenerative insults than those in males.

As noted in the Introduction section, mitochondrial dysfunction has been implicated as a potential contributor to PD pathogenesis (40). Because mitochondria are inherited exclusively through the maternal line, significant sex-related differences in mitochondrial function exist. Several studies have shown that female neurons exhibit higher electron transport chain activity and greater functional capacity than male neurons (41–43). As a result, brain mitochondria in females generally display lower levels of oxidative stress and damage compared to those in males (41, 44).

Another factor contributing to differences between male and female brains may be sex-dependent variations in inflammatory responses. This is particularly relevant because neuroinflammation is recognized as a key component in the pathogenesis of neurodegenerative diseases, including PD. In rodents, there is strong evidence of sex-related differences in microglia, including variations in cell number, morphology, and gene expression (45–47). Similar differences have been reported in humans (reviewed in (48)). Astrocytes also exhibit sex-dependent characteristics under both physiological and pathological conditions. For instance, astrocytes in males produce higher levels of IL6, TNF, and IL1β in response to LPS, whereas female astrocytes generate higher levels of interferon-inducible protein 10 (49). These disparities likely contribute to sex-specific differences in neuroinflammatory responses.

Sex-related differences in the blood-brain barrier (BBB) have also been reported, with BBB dysfunction implicated in the development and progression of neurodegenerative diseases (50).

Furthermore, studies in both animal models and humans indicate that sex can influence the gut-brain axis, as well as the effects of probiotic and prebiotic interventions on brain function and behavior (51).

Given these findings, it is evident that multiple physiological and molecular mechanisms may contribute to the greater resilience of females in our UC model. This raises the question: what underlies these sex differences? A likely explanation is the primary distinction between males and females: the differential production of sex hormones. In this context, estrogens produced by females may play a key role in many of the differences described above. Supporting this hypothesis, oophorectomy has been significantly associated with an increased incidence of parkinsonism (52). Furthermore, the neuroprotective effects of estrogens have been extensively documented (53–59).

In the SN and striatum, glial cells express both alpha and beta estrogen receptors, as well as the G-protein-coupled estrogen receptor in the striatum. Estrogens have been shown to exert neuroprotective effects in both clinical studies and experimental animal models (60–63). Mechanistically, estrogens signal through the mitogen-activated protein kinase pathway to protect against glutamate-induced neurotoxicity (64), upregulate B-cell lymphoma 2 to mitigate oxidative stress (65), and act synergistically with glutathione to scavenge free radicals (66, 67). Moreover, estradiol profoundly influences dopamine metabolism by enhancing dopamine synthesis, release, turnover, and reuptake (37, 68).

Moreover, sex hormones are known to underlie differences in microglia biology between males and females (48). These hormonal effects are particularly relevant in inflammatory and neurodegenerative contexts, where they modulate microglial function and contribute to sex-specific outcomes in neurodegenerative diseases. In this regard, estrogens have been shown to protect against neuronal death by promoting a cytoprotective microglial phenotype in the SN (69).

As noted earlier, mitochondrial dysfunction is a hallmark of PD, and sex-related differences have been observed in this context. Estrogens, which are synthesized within mitochondria, appear to play a significant role in regulating mitochondrial function. Steroid hormones, including estrogens, act on mitochondria to stimulate various processes, notably mitochondrial biogenesis. Estradiol, in particular, has been shown to prevent mitochondrial loss in aging females (70).

The integrity of the BBB, another factor relevant to PD, also appears to be modulated by estrogens. Specifically, estrogen treatment protects against inflammation-induced BBB disruption (57) and reduces leukocyte extravasation across the BBB (71).

Collectively, these findings suggest that estrogens in females, through their anti-inflammatory and neuroprotective effects, as well as their actions on mitochondria, the gut-brain axis, and the BBB, may protect against the pathogenic accumulation of α-syn, neuroinflammation, and neurodegeneration observed in the male model. While this explanation is the most plausible, it does not exclude other possibilities. For instance, Fang et al. (2024) proposed that these sex differences could also be influenced by genes located on the Y chromosome in males (72). Because estrogen levels were not directly measured in this study, any interpretations regarding their role remain speculative. Follow-up experiments, such as ovariectomy or hormone-replacement studies, will be necessary to confirm these findings.

In summary, our findings demonstrate a sex-dependent vulnerability of SNpc neurons in response to gut inflammation, highlighting the importance of accounting for sex differences in the experimental studies using PD animal models. Although female rats did not exhibit dopaminergic neuron loss in the SN, the presence of subclinical pathological changes cannot be entirely excluded. Future studies employing whole-brain imaging or proteomic analyses of additional regions implicated in early PD pathology, such as the dorsal motor nucleus of the vagus or the amygdala, could further substantiate our conclusions.

Growing evidence indicates that PD manifests differently in men and women, both in terms of symptoms and the underlying mechanisms of pathogenesis. Despite this, research addressing these sex differences remains limited in both clinical and preclinical studies, which often do not consistently account for the sex of the subjects or models used.

Studies of sex differences in the human gut-brain axis are particularly scarce. However, animal studies, including the presented work, demonstrate a significant influence of sex on this axis. Our findings highlight the importance of including representative samples of both sexes, and across the gender spectrum, in clinical studies and animal models of PD to better understand how biological, social, and environmental factors interact with the gut-brain axis. Developing therapeutic strategies tailored to both men and women, and designing clinical trials that account for these differences, is therefore essential.

In conclusion, sex differences may significantly influence PD pathogenesis. While much remains to be learned about these sex-based variations, it is clear that the gut-brain axis plays a critical role in brain function and mental health. Investigating how sex impacts this axis may improve our understanding of, and therapeutic approaches to, disorders such as PD.

## Data Availability

The raw data supporting the conclusions of this article will be made available by the authors, without undue reservation.

## References

[1] Surguchov A Surguchev A . Synucleins: new data on misfolding, aggregation and role in diseases. Biomedicines. (2022) 10:3241. doi: 10.3390/biomedicines10123241, PMID: 36551997 PMC9775291

[2] Dauer W Przedborski S . Parkinson’s disease: mechanisms and models. Neuron. (2003) 39:889–909. doi: 10.1016/S0896-6273(03)00568-3, PMID: 12971891

[3] Hunot S Hirsch EC . Neuroinflammatory processes in Parkinson’s disease. Ann Neurol. (2003) 53:S49–60. doi: 10.1002/ana.10481, PMID: 12666098

[4] Dexter DT Jenner P . Parkinson disease: from pathology to molecular disease mechanisms. Free Radic Biol Med. (2013) 62:132–44. doi: 10.1016/j.freeradbiomed.2013.01.018, PMID: 23380027

[5] Feigin VL Nichols E Alam T Bannick MS Beghi E Blake N . Global, regional, and national burden of neurological disorders, 1990&x2013;2016: a systematic analysis for the Global Burden of Disease Study 2016. Lancet Neurol. (2019) 18:459–80. doi: 10.1016/S1474-4422(18)30499-X, PMID: 30879893 PMC6459001

[6] Chen QQ Haikal C Li W Li JY . Gut inflammation in association with pathogenesis of parkinson’s disease. Front Mol Neurosci. (2019) 12:218. doi: 10.3389/fnmol.2019.00218, PMID: 31572126 PMC6753187

[7] Zhu F Li C Gong J Zhu W Gu L Li N . The risk of Parkinson’s disease in inflammatory bowel disease: A systematic review and meta-analysis. Dig Liver Dis. (2019) 51:38–42. doi: 10.1016/j.dld.2018.09.017, PMID: 30309751

[8] Kim GH Lee YC Kim TJ Kim ER Hong SN Chang DK . Risk of neurodegenerative diseases in patients with inflammatory bowel disease: A nationwide population-based cohort study. J Crohns Colitis. (2022) 16:436–43. doi: 10.1093/ecco-jcc/jjab162, PMID: 34499125

[9] Espinosa-Oliva AM Ruiz R Soto MS Boza-Serrano A Rodriguez-Perez AI Roca-Ceballos MA . Inflammatory bowel disease induces pathological α-synuclein aggregation in the human gut and brain. Neuropathol Appl Neurobiol. (2024) 50:e12962. doi: 10.1111/nan.12962, PMID: 38343067

[10] Crispino P Gino M Barbagelata E Ciarambino T Politi C Ambrosino I . Gender differences and quality of life in parkinson’s disease. Int J Environ Res Public Health. (2020) 18:198. doi: 10.3390/ijerph18010198, PMID: 33383855 PMC7795924

[11] Houser MC Caudle WM Chang J Kannarkat GT Yang Y Kelly SD . Experimental colitis promotes sustained, sex-dependent, T-cell-associated neuroinflammation and parkinsonian neuropathology. Acta Neuropathol Commun. (2021) 9:139. doi: 10.1186/s40478-021-01240-4, PMID: 34412704 PMC8375080

[12] Russillo MC Andreozzi V Erro R Picillo M Amboni M Cuoco S . Sex differences in parkinson’s disease: from bench to bedside. Brain Sci. (2022) 12:917. doi: 10.3390/brainsci12070917, PMID: 35884724 PMC9313069

[13] Okayasu I Hatakeyama S Yamada M Ohkusa T Inagaki Y Nakaya R . A novel method in the induction of reliable experimental acute and chronic ulcerative colitis in mice. Gastroenterology. (1990) 98:694–702. doi: 10.1016/0016-5085(90)90290-H, PMID: 1688816

[14] García-Revilla J Boza-Serrano A Jin Y Vadukul DM Soldán-Hidalgo J Camprubí-Ferrer L . Galectin-3 shapes toxic alpha-synuclein strains in Parkinson’s disease. Acta Neuropathol. (2023) 146:51–75. doi: 10.1007/s00401-023-02585-x, PMID: 37202527 PMC10261194

[15] Cooper HS Murthy SNS Shah RS Sedergran DJ . Clinicopathologic study of dextran sulfate sodium experimental murine colitis. Lab Invest. (1993) 69:238–49., PMID: 8350599

[16] Gundersen HJG Bagger P Bendtsen TF Evans SM Korbo L Marcussen N . The new stereological tools: Disector, fractionator, nucleator and point sampled intercepts and their use in pathological research and diagnosis. APMIS. (1988) 96:857–81. doi: 10.1111/j.1699-0463.1988.tb00954.x, PMID: 3056461

[17] Paxinos G Watson C . The rat in stereotaxic coordinates. San Diego, CA: Academic Press (1986).

[18] Villarán RF Espinosa-Oliva AM Sarmiento M De Pablos RM Argüelles S Delgado-Cortés MJ . Ulcerative colitis exacerbates lipopolysaccharide-induced damage to the nigral dopaminergic system: potential risk factor in Parkinson`s disease. J Neurochem. (2010) 114:1687–700. doi: 10.1111/j.1471-4159.2010.06879.x, PMID: 20584104

[19] MaChado A Herrera AJ Venero JL Santiago M De Pablos RM Villarán RF . Peripheral inflammation increases the damage in animal models of nigrostriatal dopaminergic neurodegeneration: possible implication in Parkinson’s disease incidence. Parkinsons Dis. (2011) 2011:393769. doi: 10.4061/2011/393769, PMID: 21603178 PMC3096050

[20] Hernández-Romero MC Delgado-Cortés MJ Sarmiento M de Pablos RM Espinosa-Oliva AM Argüelles S . Peripheral inflammation increases the deleterious effect of CNS inflammation on the nigrostriatal dopaminergic system. Neurotoxicology. (2012) 33:347–60. doi: 10.1016/j.neuro.2012.01.018, PMID: 22330755

[21] Casini A Vivacqua G Ceci L Leone S Vaccaro R Tagliafierro M . TNBS colitis induces architectural changes and alpha-synuclein overexpression in mouse distal colon: A morphological study. Cell Tissue Res. (2025) 399:247–65. doi: 10.1007/s00441-024-03932-4, PMID: 39656240 PMC11787265

[22] Cerri S Mus L Blandini F . Parkinson’s disease in women and men: what’s the difference? J Parkinsons Dis. (2019) 9:501–15. doi: 10.3233/JPD-191683, PMID: 31282427 PMC6700650

[23] Bábíčková J Tóthová L Lengyelová E Bartoňová A Hodosy J Gardlík R . Sex differences in experimentally induced colitis in mice: a role for estrogens. Inflammation. (2015) 38:1996–2006. doi: 10.1007/s10753-015-0180-7, PMID: 25962374

[24] Wagnerova A Babickova J Liptak R Vlkova B Celec P Gardlik R . Sex differences in the effect of resveratrol on DSS-induced colitis in mice. Gastroenterol Res Pract. (2017) 2017:8051870. doi: 10.1155/2017/8051870, PMID: 28465680 PMC5390549

[25] Fang X Yang J Yang L Lin Y Li Y Yin X . Multi-omics analysis identified macrophages as key contributors to sex-related differences in ulcerative colitis. Front Immunol. (2025) 16:1569271. doi: 10.3389/fimmu.2025.1569271, PMID: 40636118 PMC12238219

[26] Perryman LE Wyatt CR Magnuson NS Mason PH . T lymphocyte development and maturation in horses. Anim Genet. (1988) 19:343–8. doi: 10.1111/j.1365-2052.1988.tb00825.x, PMID: 3069010

[27] Cox LM Abou-El-Hassan H Maghzi AH Vincentini J Weiner HL . The sex-specific interaction of the microbiome in neurodegenerative diseases. Brain Res. (2019) 1724:146385. doi: 10.1016/j.brainres.2019.146385, PMID: 31419428 PMC6886714

[28] Costa G Porceddu PF Serra M Casu MA Schiano V Napolitano F . Lack of rhes increases mdma-induced neuroinflammation and dopamine neuron degeneration: Role of gender and age. Int J Mol Sci. (2019) 20:1556. doi: 10.3390/ijms20071556, PMID: 30925704 PMC6480667

[29] Seyfried TN Choi H Chevalier A Hogan D Akgoc Z Schneider JS . Sex-related abnormalities in substantia nigra lipids in parkinson’s disease. ASN Neuro. (2018) 10:1759091418781889. doi: 10.1177/1759091418781889, PMID: 29932343 PMC6024349

[30] Hadler NM . Regional musculoskeletal diseases of the low back. Cumulative trauma versus single incident. Clin Orthop Relat Res. (1987) (221):33–41. doi: 10.1097/00003086-198708000-00005, PMID: 2955988

[31] Smith KM Dahodwala N . Sex differences in Parkinson’s disease and other movement disorders. Exp Neurol. (2014) 259:44–56. doi: 10.1016/j.expneurol.2014.03.010, PMID: 24681088

[32] Bourque M Morissette M Di Paolo T . Neuroactive steroids and Parkinson’s disease: Review of human and animal studies. Neurosci Biobehav Rev. (2024) 156:105479. doi: 10.1016/j.neubiorev.2023.105479, PMID: 38007170

[33] Silva RH Lopes-Silva LB Cunha DG Becegato M Ribeiro AM Santos JR . Animal approaches to studying risk factors for parkinson’s disease: A narrative review. Brain Sci. (2024) 14:156. doi: 10.3390/brainsci14020156, PMID: 38391730 PMC10887213

[34] Cantuti-Castelvetri I Keller-McGandy C Bouzou B Asteris G Clark TW Frosch MP . Effects of gender on nigral gene expression and parkinson disease. Neurobiol Dis. (2007) 26:606–14. doi: 10.1016/j.nbd.2007.02.009, PMID: 17412603 PMC2435483

[35] Simunovic F Yi M Wang Y Stephens R Sonntag KC . Evidence for gender-specific transcriptional profiles of nigral dopamine neurons in Parkinson Disease. PloS One. (2010) 5:e8856. doi: 10.1371/journal.pone.0008856, PMID: 20111594 PMC2810324

[36] Pohjalainen T Rinne JO Någren K Syvälahti E Hietala J . Sex differences in the striatal dopamine D2 receptor binding characteristics *in vivo*. Am J Psychiatry. (1998) 155:768–73. doi: 10.1176/ajp.155.6.768, PMID: 9619148

[37] Mariani E Lombardini L Facchin F Pizzetti F Frabetti F Tarozzi A . Sex-specific transcriptome differences in substantia Nigra tissue: A meta-analysis of parkinson’s disease data. Genes (Basel). (2018) 9:275. doi: 10.3390/genes9060275, PMID: 29799491 PMC6027313

[38] Cullity ER Madsen HB Perry CJ Kim JH . Postnatal developmental trajectory of dopamine receptor 1 and 2 expression in cortical and striatal brain regions. J Comp Neurology. (2019) 527:1039–55. doi: 10.1002/cne.24574, PMID: 30408161

[39] Pinares-Garcia P Stratikopoulos M Zagato A Loke H Lee J . Sex: A significant risk factor for neurodevelopmental and neurodegenerative disorders. Brain Sci. (2018) 8:154. doi: 10.3390/brainsci8080154, PMID: 30104506 PMC6120011

[40] Malpartida AB Williamson M Narendra DP Wade-Martins R Ryan BJ . Mitochondrial dysfunction and mitophagy in parkinson’s disease: from mechanism to therapy. Trends Biochem Sci. (2021) 46:329–43. doi: 10.1016/j.tibs.2020.11.007, PMID: 33323315

[41] Gaignard P Savouroux S Liere P Pianos A Thérond P Schumacher M . Effect of sex differences on brain mitochondrial function and its suppression by ovariectomy and in aged mice. Endocrinology. (2015) 156:2893–904. doi: 10.1210/en.2014-1913, PMID: 26039154

[42] Escames G Díaz-Casado ME Doerrier C Luna-Sánchez M López LC Acuña-Castroviejo D . Early gender differences in the redox status of the brain mitochondria with age: effects of melatonin therapy. Horm Mol Biol Clin Investig (2013) 16:91–100. doi: 10.1515/hmbci-2013-0026, PMID: 25436750

[43] Harish G Venkateshappa C Mahadevan A Pruthi N Bharath MMS Shankar SK . Mitochondrial function in human brains is affected by pre- and post mortem factors. Neuropathol Appl Neurobiol. (2013) 39:298–315. doi: 10.1111/j.1365-2990.2012.01285.x, PMID: 22639898

[44] Guevara R Gianotti M Oliver J Roca P . Age and sex-related changes in rat brain mitochondrial oxidative status. Exp Gerontol. (2011) 46:923–8. doi: 10.1016/j.exger.2011.08.003, PMID: 21864669

[45] Han J Fan Y Zhou K Blomgren K Harris RA . Uncovering sex differences of rodent microglia. J Neuroinflammation. (2021) 18:74. doi: 10.1186/s12974-021-02124-z, PMID: 33731174 PMC7972194

[46] Nelson LH Lenz KM . The immune system as a novel regulator of sex differences in brain and behavioral development. J Neurosci Res. (2017) 95:447–61. doi: 10.1002/jnr.23821, PMID: 27870450 PMC8008603

[47] Villa A Gelosa P Castiglioni L Cimino M Rizzi N Pepe G . Sex-specific features of microglia from adult mice. Cell Rep. (2018) 23:3501–11. doi: 10.1016/j.celrep.2018.05.048, PMID: 29924994 PMC6024879

[48] Bourque M Morissette M Soulet D Di Paolo T . Impact of sex on neuroimmune contributions to Parkinson’s disease. Brain Res Bull. (2023) 199:110668. doi: 10.1016/j.brainresbull.2023.110668, PMID: 37196734

[49] Santos-Galindo M Acaz-Fonseca E Bellini MJ Garcia-Segura LM . Sex differences in the inflammatory response of primary astrocytes to lipopolysaccharide. Biol Sex Differ. (2011) 2:7. doi: 10.1186/2042-6410-2-7, PMID: 21745355 PMC3143074

[50] Sohrabji F . Guarding the blood-brain barrier: A role for estrogen in the etiology of neurodegenerative disease. Gene Expression. (2007) 13:311–9. doi: 10.3727/000000006781510723, PMID: 17708417 PMC6032455

[51] Holingue C Budavari AC Rodriguez KM Zisman CR Windheim G Fallin MD . Sex differences in the gut-brain axis: implications for mental health. Curr Psychiatry Rep. (2020) 22:83. doi: 10.1007/s11920-020-01202-y, PMID: 33216233 PMC7717677

[52] Ali A Tabassum SA Rehman Z Ramani M Ali K Siddiqui AM . Association of bilateral oophorectomy with incidence of Parkinson’s disease: A systematic review and meta-analysis. Parkinsonism Relat Disord. (2024) 121:106025. doi: 10.1016/j.parkreldis.2024.106025, PMID: 38364624

[53] Dluzen DE McDermott JL Liu B . Estrogen as a neuroprotectant against MPTP-induced neurotoxicity in C57/B1 mice. Neurotoxicol Teratol. (1996) 18:603–6. doi: 10.1016/0892-0362(96)00086-4, PMID: 8888025

[54] Disshon KA Dluzen DE . Estrogen as a neuromodulator of MPTP-induced neurotoxicity: effects upon striatal dopamine release. Brain Res. (1997) 764:9–16. doi: 10.1016/s0006-8993(97)00418-6, PMID: 9295188

[55] Sawada H Ibi M Kihara T Urushitani M Honda K Nakanishi M . Mechanisms of antiapoptotic effects of estrogens in nigral dopaminergic neurons. FASEB J. (2000) 14:1202–14. doi: 10.1096/fasebj.14.9.1202, PMID: 10834942

[56] Shulman LM . Is there a connection between estrogen and Parkinson’s disease? Parkinsonism Relat Disord. (2002) 8:289–95. doi: 10.1016/s1353-8020(02)00014-7, PMID: 15177058

[57] Tomás-Camardiel M Venero JL Herrera AJ De Pablos RM Pintor-Toro JA MaChado A . Blood–brain barrier disruption highly induces aquaporin-4 mRNA and protein in perivascular and parenchymal astrocytes: Protective effect by estradiol treatment in ovariectomized animals. J Neurosci Res. (2005) 80:235–46. doi: 10.1002/jnr.20443, PMID: 15772982

[58] Villa A Vegeto E Poletti A Maggi A . Estrogens, neuroinflammation, and neurodegeneration. Endocr Rev. (2016) 37:372–402. doi: 10.1210/er.2016-1007, PMID: 27196727 PMC4971309

[59] Thadathil N Xiao J Hori R Alway SE Khan MM . Brain selective estrogen treatment protects dopaminergic neurons and preserves behavioral function in MPTP-induced mouse model of parkinson’s disease. J Neuroimmune Pharmacol. (2021) 16:667–78. doi: 10.1007/s11481-020-09972-1, PMID: 33221984 PMC8140060

[60] Brann DW Dhandapani K Wakade C Mahesh VB Khan MM . Neurotrophic and neuroprotective actions of estrogen: Basic mechanisms and clinical implications. Steroids. (2007) 72:381–405. doi: 10.1016/j.steroids.2007.02.003, PMID: 17379265 PMC2048656

[61] Raghava N Das BC Ray SK . Neuroprotective effects of estrogen in CNS injuries: insights from animal models. Neurosci Neuroecon. (2017) 6:15–29. doi: 10.2147/NAN.S105134, PMID: 28845391 PMC5567743

[62] Zárate S Stevnsner T Gredilla R . Role of estrogen and other sex hormones in brain aging. Neuroprotection and DNA repair. Front Aging Neurosci. (2017) 9. doi: 10.3389/fnagi.2017.00430, PMID: 29311911 PMC5743731

[63] Inestrosa NC Marzolo MP Bonnefont AB . Cellular and molecular basis of estrogen’s neuroprotection. Mol Neurobiol. (1998) 17:73–86. doi: 10.1007/BF02802025, PMID: 9887447

[64] Singer CA Figueroa-Masot XA Batchelor RH Dorsa DM . The mitogen-activated protein kinase pathway mediates estrogen neuroprotection after glutamate toxicity in primary cortical neurons. J Neurosci. (1999) 19:2455. doi: 10.1523/JNEUROSCI.19-07-02455.1999, PMID: 10087060 PMC6786088

[65] Singer CA Rogers KL Dorsa DM . Modulation of Bcl-2 expression: a potential component of estrogen protection in NT2 neurons. Neuroreport. (1998) 9:2565–8. doi: 10.1097/00001756-199808030-00025, PMID: 9721933

[66] Green PS Gridley KE Simpkins JW . Nuclear estrogen receptor-independent neuroprotection by estratrienes: a novel interaction with glutathione. Neuroscience. (1998) 84:7–10. doi: 10.1016/S0306-4522(97)00595-2, PMID: 9522357

[67] Wooten GF Currie LJ Bovbjerg VE Lee JK Patrie J . Are men at greater risk for Parkinson’s disease than women? J Neurology Neurosurg Psychiatry. (2004) 75:637. doi: 10.1136/jnnp.2003.020982, PMID: 15026515 PMC1739032

[68] Lee JJ Ham JH Lee PH Sohn YH . Gender differences in age-related striatal dopamine depletion in parkinson’s disease. J Mov Disord. (2015) 8:130–5. doi: 10.14802/jmd.15031, PMID: 26413240 PMC4572663

[69] Siani F Greco R Levandis G Ghezzi C Daviddi F Demartini C . Influence of estrogen modulation on glia activation in a murine model of parkinson’s disease. Front Neurosci. (2017) 11:306. doi: 10.3389/fnins.2017.00306, PMID: 28620274 PMC5449471

[70] Waters EM Mazid S Dodos M Puri R Janssen WG Morrison JH . Effects of estrogen and aging on synaptic morphology and distribution of phosphorylated Tyr1472 NR2B in the female rat hippocampus. Neurobiol Aging. (2019) 73:200–10. doi: 10.1016/j.neurobiolaging.2018.09.025, PMID: 30384123 PMC11548941

[71] Maggioli E McArthur S Mauro C Kieswich J Kusters DHM Reutelingsperger CPM . Estrogen protects the blood–brain barrier from inflammation-induced disruption and increased lymphocyte trafficking. Brain Behav Immun. (2016) 51:212–22. doi: 10.1016/j.bbi.2015.08.020, PMID: 26321046

[72] Fang P Yu LW Espey H Agirman G Kazmi SA Li K . Sex-dependent interactions between prodromal intestinal inflammation and LRRK2 G2019S in mice promote endophenotypes of Parkinson’s disease. Commun Biol. (2024) 7:570. doi: 10.1038/s42003-024-06256-9, PMID: 38750146 PMC11096388

